# Retraction: Mathematical modeling and numerical simulation of supercritical processing of drug nanoparticles optimization for green processing: AI analysis

**DOI:** 10.1371/journal.pone.0316403

**Published:** 2024-12-19

**Authors:** The PLOS ONE Editors

The *PLOS ONE* Editors retract this article [1] because it was identified as one of a series of submissions for which we have concerns about potential manipulation of the publication process and authorship. These concerns call into question the validity and provenance of the reported results. We regret that the issues were not identified prior to the article’s publication.

The corresponding author acknowledged that they used a writing and language service to assist with the preparation and submission of the manuscript and supporting files. A representative of the service confirmed that they edited and submitted the manuscript, and stated that they did not contribute to the content. This information did not resolve the concerns regarding potential manipulation of the publication process and authorship.

The corresponding author reported that their ORCID number is incorrect on the published article as it should be 0000-0003-0696-302X.

The author did not agree with the retraction.
